# Dance intervention targeting motor, cognitive, and emotional components in stroke patients and their caregivers: a feasibility study

**DOI:** 10.3389/fpsyg.2026.1872042

**Published:** 2026-07-27

**Authors:** Mateja Virovec, Gloria Massironi, Nicola Refatti, Gaspare Crimi, Marcella Galbusera, Michela Rimondini, Silvia Savazzi, Valeria Donisi, Paola Cesari

**Affiliations:** 1 Department of Neuroscience, Biomedicine and Movement Sciences, University of Verona, Verona, Italy; 2 Physical Medicine and Rehabilitation Unit, Marzana Hospital, Verona, Italy; 3 Independent Researcher, Verona, Italy

**Keywords:** caregivers, dance and movement, feasibility studies, psychosocial wellbeing, stroke

## Abstract

**Introduction:**

This study aimed to co-create and assess the feasibility of a novel, museum-based contemporary dance intervention for post-stroke patients and their informal caregivers. The study explored the intervention's feasibility, safety, and preliminary effects on motor, cognitive, and psychosocial wellbeing for both patients and caregivers.

**Methods:**

A mixed-methods design was employed, starting with a co-creation focus group (*n* = 4 patients, *n* = 3 caregivers). This was followed by a single-arm, pre-post feasibility pilot. A total of 16 participants were recruited from a hospital rehabilitation unit. The final analyzed sample consisted of four patients and four caregivers. The intervention consisted of weekly 60-min dance classes conducted in a museum setting over 20 weeks. Feasibility indicators included recruitment/retention rates, attendance, and adverse events. Quantitative assessments of motor function, global cognition, and psychosocial wellbeing (burden and quality of life) were conducted at baseline and at 20 weeks.

**Results:**

The adapted dance intervention was highly acceptable with high attendance and satisfaction. In exploratory analyses, both patients and caregivers exhibited positive trends in global cognition. Caregivers showed slight improvements in wellbeing, though subjective burden remained unchanged. Descriptive observations suggested a potential decrease in gait imbalance among patients, while standing balance and spatiotemporal parameters remained stable. No adverse events were reported.

**Discussion:**

Preliminary evidence indicates a positive tendency toward the feasibility and acceptability of this dance intervention as a community-based tool for stroke rehabilitation. The museum setting and the dyadic nature of the intervention provide a promising environment for improving cognitive and motor outcomes. These preliminary findings indicate that integrating dance into rehabilitation shows promise in terms of safety and potential benefits for both patients and caregivers, warranting further investigation in a larger controlled trial.

## Introduction

Globally, stroke is the leading cause of acquired disability and the second leading cause of death, right after ischemic heart disease (Feigin et al., 2017). As most stroke patients survive the initial stage of the illness, the biggest health impact is typically caused by the long-term consequences affecting both patients and their families (Langhorne et al., 2011). Stroke can result in physical, cognitive, emotional, behavioral, and perceptive impairments (Mukherjee et al., 2006; Rost et al., 2022). Physical impairments can cause difficulties with balance, gait, and mobility (Langhorne et al., 2009), while cognitive impairments, affecting over 70% of stroke patients (Wagle et al., 2011), can impact memory, executive functioning, attention, language, and visuospatial abilities (Jokinen et al., 2015). These deficits often lead to psychological distress (Crowe et al., 2016), social isolation (Salter et al., 2008), and reduced quality of life (Schmid et al., 2013).

Furthermore, the burden also extends to informal caregivers, in most cases, spouses who take the role of the primary caregiver, thus being identified as the group most at risk of experiencing stress and burden (Rigby et al., 2009). Caring for a family member involves both physical and emotional assistance. Consequently, higher rates of anxiety (Zarit and Edwards, 1996), substantial limitations in social activities (Velloze et al., 2022) and a lower quality of life were reported in family caregivers looking after stroke survivors. Nevertheless, informal caregivers frequently remain an overlooked population, often excluded from recovery support protocols.

Current practice in stroke focuses on functional recovery (Li et al., 2024), yet dance offers broader benefits by integrating physical (Keogh et al., 2009; Liu et al., 2021), cognitive (Foster, 2013), and social engagement with musical experience (Kattenstroth et al., 2010). Its efficacy is already well-established in other neurological populations, such as dementia (Klimova et al., 2017) and Parkinson's disease (Hackney and Earhart, 2009). While qualitative evidence suggests that the social nature of dance is crucial for fostering emotional security (Kipnis et al., 2023), stroke research remains limited by inconsistent protocols and a primary focus on motor outcomes. Additionally, existing studies often utilize pre-defined dance styles (e.g., tango) that have reported high participant satisfaction (Hackney et al., 2012; Rochetti et al., 2021) and increased wellbeing (Varga et al., 2023); however these interventions have overlooked participant-centered design.

Recently, participatory approaches have gained more attention as a standard approach in health research, emphasizing partnerships between researchers and communities to improve intervention design (Bensing et al., 2013) through a co-creation process. This process creates tailored interventions that improve adherence, particularly for chronic diseases (Donisi et al., 2022).

Building on previous research, the present study utilized a co-creation design to develop a dance intervention tailored to the needs of stroke survivors and their informal caregivers. Contemporary dance was selected because it emphasizes adaptability, creativity, individual movement exploration, and expression rather than pre-defined movement sequences. These characteristics may be particularly suitable for heterogeneous stroke populations with varying physical and cognitive abilities, a versatility already observed in its application with other neurological disorders, specifically Parkinson's disease (Volpe et al., 2025). Unlike partner-based forms such as tango or ballroom dance, contemporary dance allows movements to be individualized while maintaining opportunities for social interaction and artistic engagement.

Recognizing the critical yet often overlooked role of the caregiver in stroke recovery, our primary aim was to engage both stroke survivors and caregivers in the program's creation. By integrating their needs and inputs, we sought to develop an intervention that was not only appropriate for patients but also supportive and accessible for their caregivers. We then tested the feasibility of the intervention, with secondary aims including the assessment of preliminary motor, cognitive, and psychosocial outcomes, alongside overall participant satisfaction.

## Methods

### Study design

This study was carried out according to the ethical principles outlined in the 1964 Declaration of Helsinki (World Medical Association World Medical Association., 2025) and was approved by the Regional Ethics Committee for the South-West Area of Veneto (CET-ASOV): Prog. 432CET. All participants read and signed informed consent. A mixed-methods feasibility design was employed. The co-creation phase (phase 1) consisted of a focus group discussion to define the intervention protocols, and the intervention phase (phase 2) utilized a single-arm pre-post design to evaluate the feasibility and preliminary impact of the dance intervention. The assessments were conducted at baseline (week 1) and post-intervention (week 20).

### Participants

From November 2024 to January 2025, adults who were clinically diagnosed with ischemic or hemorrhagic stroke at least 6 months prior, and their primary caregivers, were recruited from a Physical Medicine and Rehabilitation Unit of Marzana Hospital (Verona, Italy). Potential participants were identified and screened by a neurologist (NR) and a neuropsychologist (GM) for both phases 1 and 2. Stroke survivors were eligible if they were aged ≥18 years, had a clinical diagnosis of ischemic or hemorrhagic stroke ≥6 months prior (chronic phase), provided written informed consent, and demonstrated sufficient physical and cognitive capacity to tolerate the suggested activities and follow study procedures. Caregivers were included if they were the primary unpaid provider of support for the patient and provided informed consent. Individuals were excluded if they presented severe hearing or visual impairments, comorbid neurological, orthopedic, or rheumatic disorders interfering with physical activity, or unstable cardiovascular conditions.

#### Phase 1: focus group

A focus group discussion was conducted to explore the perspectives and preferences of stroke survivors and caregivers regarding the acceptability and perceived relevance of a planned dance-based intervention. A semi-structured question guide with open-ended questions was used (see Supplementary appendix S1). Prior to the session, all participants completed a questionnaire to collect socio-demographic and clinical information. The focus group was conducted in January 2025 and moderated by two clinical psychologists (MR and VD), with two additional researchers acting as observers (MV and PC). Participants were invited to share their experiences, opinions, and insights to inform their understanding of the feasibility and contextual suitability of the intervention. The discussion lasted approximately 1.5 h.

#### Phase 2: intervention

The dance-based intervention was grounded in the established Dance Well program, an activity originally developed for people with Parkinson's disease and other movement disorders to promote social inclusion and wellbeing (Comune di Bassano del Grappa, 2026) (see Supplementary appendix 2 for a full description). Core methodological components were pre-determined prior to the focus group discussions, including: the use of contemporary dance as the activity modality, the cultural venue setting (*Museo degli Affreschi “G.B. Cavalcaselle” alla Tomba di Giulietta* in Verona, Italy), which is a distinctive feature of the Dance Well program designed to enhance the artistic experience, and the basic class structure and instructor qualifications. The dance sessions were held once per week (20 weeks total) in the morning hours. Each session lasted 60 min, and they were free of charge. The sessions were led by a professional dancer and teacher with over 30 years of experience teaching contemporary dance and 5 years of experience working with individuals diagnosed with Parkinson's disease. An assistant dance teacher, who is also a physiotherapist, was present during the sessions to provide additional support. The intervention was designed as an inclusive group activity rather than a strictly dyadic partner-dance, allowing stroke survivors and caregivers to participate simultaneously as equals while freely exploring movements within the space. While both groups received identical instructions, the experience was meant to be flexible, allowing all participants to adapt the creative improvisation to their unique physical, emotional, and cognitive needs. In line with the principles of Dance Well, this approach emphasizes individual artistic expression and shared somatic awareness. Accordingly, while a stroke survivor might focus on exploring movement boundaries within motor limitations, a caregiver might engage with the same exercise for physical release. Ultimately, the goal is that both groups find a shared space for psychosocial release, emotional expression, and mutual support.

### Assessments

Participants were tested 1 week before the start of the intervention (baseline: T0) and after the 20-week dance intervention (post-intervention: T1) using a battery of questionnaires.

#### Cognitive assessment (patients and caregivers)

Global cognitive function was assessed both in patients and caregivers using the Italian version of the Montreal Cognitive Assessment (MoCA) (Siciliano et al., 2019), which is considered a good predictor of mild post-stroke cognitive impairment (Salvadori et al., 2013). Memory was evaluated via Digit Span Forward and Backwards (short-term and working memory) (Monaco et al., 2013) and the Italian version of the Short Story Recall test (long-term memory) (Spinnler and Tognoni, 1987). Executive function and visual attention were measured using the Trail Making Test (Parts A and B) adapted to the Italian sample (Siciliano et al., 2019).

#### Motor assessment (patients and caregivers)

Gait analysis was performed using the OptoJump Next System (Microgate, Italy), an optical measurement system consisting of a transmitting and receiving bar that acquires the fundamental parameters of gait. The variables analyzed included gait velocity, cadence, asymmetry, and both step and stride length. Data were derived by averaging the results from three trials conducted in each direction. Postural stability was evaluated using a standard force plate (OR-5, AMTI, Watertown, MA; 90 × 90 cm) during a 30-second quiet standing task. Center of pressure (CoP) displacement was recorded under two sensory conditions: eyes open and eyes closed. For each condition, the total distance traveled by the CoP (total length), the average speed of CoP displacement (total velocity), and the 95% confidence ellipse area covering the CoP trajectory (sway area) were calculated.

#### Patient-reported outcomes

Patients completed two self-administered questionnaires: the Italian version of the Hospital Anxiety and Depression Scale (HADS) (Costantini et al., 1999; Zigmond and Snaith, 1983), specifically created for patients with a physical illness and validated for use in stroke survivors, and the Italian version of the Brief Illness Perception Questionnaire (B-IPQ) (Broadbent et al., 2006; Pain et al., 2006).

#### Caregiver-reported outcomes

Caregivers completed the Italian version of the Zarit Burden Interview (ZBI) (Chattat et al., 2011), a questionnaire measuring caregivers' subjective burden of providing care to chronically disabled persons. To assess caregivers' general wellbeing, the Five-Item World Health Organization Wellbeing Index (WHO-5) was administered (Bonomi et al., 2000).

#### Satisfaction and perceived improvements (patients and caregivers)

At the post-intervention assessment, both patients and caregivers were given an Exit Questionnaire, a previously validated tool (Hackney et al., 2007) that evaluated their satisfaction with the program and perceived improvements. Participants were asked if they enjoyed participating, if they would continue with the classes if given the opportunity, and whether they found the program feasible. Items two through seven asked if participants noted improvements in particular aspects of physical wellbeing.

Data on retention and adverse effects were collected during each session throughout the intervention period. Retention was measured as a percentage of participants who completed the study, and attendance was calculated as the percentage of classes completed by all who started the intervention. Adverse effects reported during the classes or communicated to the dance instructor were tracked during each session.

## Data analysis

### Co-creation phase

Qualitative data were analyzed using directed content analysis (Hsieh and Shannon, 2005). This approach was chosen because the coding process was guided by the semi-structured interview protocol, focusing on pre-established categories including physical, psychological, and social factors, as well as organizational aspects of the intervention and venue. Audio recordings were transcribed verbatim and reviewed multiple times. Next, data segments relevant to the study objectives were identified and categorized into pre-determined themes. Finally, the text was coded into sub-themes derived directly from the participants' responses to capture specific nuances within the broader categories.

### Intervention phase

Changes in outcome measures from pre- to post-test were analyzed using JASP (Version 0.14.0.0). Due to the small sample size and the non-normal distribution of scores, within-group changes were analyzed using Wilcoxon signed-rank tests. Results are presented as means and standard deviations (SD) to maintain clinical interpretability. The median and interquartile ranges were calculated for responses to each item on the Exit Questionnaire.

## Results

### Co-creation phase

Initially, 11 individuals were recruited (six patients, five caregivers) for the discussion. Due to acute illness on the day of the session, four participants (two patients, two caregivers) were unable to attend. The final FGD comprised a purposive sample of four stroke survivors (mean age = 76.8 ± 9.9 years; three males, one female) and three caregivers (mean age = 62.7 ± 9.6 years; three females). One stroke survivor with aphasia participated; thus, specific communication accommodations, such as the extended response time and help from caregivers, were provided to ensure their perspectives were fully captured. Characteristics of the focus group participants are summarized in Supplementary Table S1.

### Focus group discussion

Three main themes were extracted through the directed content analysis: intervention components, Organizational aspects of the intervention, Obstacles and facilitators. A summary of these themes with representative quotes is presented in Table 1. Data from the focus group provided a practical validation of the intervention's logistical framework. Participants explicitly identified morning sessions as the most viable time for attendance. Furthermore, a consensus emerged regarding the timing of the program, participants expressed a strong preference for the intervention to conclude prior to August to avoid overlap with summer holidays, which were cited as a significant barrier to consistent participation.

**Table 1:** Focus group results on contents and organization of the intervention from the perspectives of patients and caregivers.

Intervention components	Themes (illustrative quotes)
Social aspects	**Sharing experiences with people who went through the same experience** The social aspect of the intervention was perceived as essential, as it fosters a sense of connection and encourages the sharing of experiences among stroke survivors and their caregivers. (Patient, male: “*Because being in a group of people, sharing this experience, is a form of personal growth. It's not like going to a café with so-called ‘normal' people. Here, everyone who joins the group has been through a similar experience.”*) **Preventing social isolation** Stroke survivors argued that this intervention might be a source of motivation to go out and socialize. (Patient, female: “*I'm doing it to socialize, to get out, to have a purpose, something to give me a reason to go out.”*) The importance of the social component was particularly emphasized by caregivers, who described a significant decline in social activities following their partners' stroke (Caregiver, female: “*Also, because, if he doesn't want to go out, what can you do, leave him home alone? So, for that very reason, what he doesn't do, you don't do either. Of course. That's just how it is.”*) Therefore, participants hoped that intervention may help prevent social isolation for both stroke survivors and caregivers. (Caregiver, female: “*I also hope that, through all of this, he will feel more willing to go out and be with others, because I'm struggling.”*)
Psychological aspects	**Improve acceptance** Participants who had suffered a stroke discussed the difficulty of accepting their current situation. They also highlighted how easily they become emotional, as they can no longer live life the way they did before the illness (Patient, male*: “The lack of normalcy. What was normal before is no longer normal. So, if we don't accept this, we get emotional right away, we cry right away.”*) Both stroke survivors and caregivers shared their hope of finding a path toward self-acceptance. (Patient, female: “*I think the first thing for us is to accept ourselves. If we accept ourselves, everything will go smoothly, in my opinion.”*). (Caregiver, female: “*In any case, by the end of this journey, all of us, even those of us who are so-called ‘normal', if we manage to accept things, then we've taken a big step forward.”)* **Reducing the stigma** Stroke survivors expressed feeling stigmatized in their day-to-day interactions due to their condition. (Patient, male: “*I feel a bit like the little monkey of the village…everyone is watching me.”*) Thus, this intervention could also help reduce feelings of judgment and social stigma, by creating a space where participants can share their experiences with others who have faced similar challenges. (Caregiver, female: “*I believe that one of the motivations could be simply being together, that is, being with people who have followed the same path.”*)
Physical aspects	**Physical improvements** Stroke survivors shared that the physical difficulties resulting from their stroke have significantly limited their daily lives. (Patient, male: “*I miss riding my bike, doing the shopping on my own.”*) As a result, they hope to see some physical improvements that could enhance their ability to carry out everyday activities (Patient, male: “*Positive changes that help, even slightly, to improve the current way of life.”)* **Relaxation** One stroke survivor mentioned that when the intervention was first introduced, she immediately associated it with slow movements that could help her relax and reduce stress (Patient, female: *then I thought about those slow movements, the kind suited for a certain age, you know. Something that really relaxes the spirit.”*) On the other hand, caregivers expressed that they hope to have fun while engaging in physical activity through dance (Caregiver, female*: “The doctor told me: I think you're going to have more fun.”*)
Organizational aspects of the intervention	
Venue	All participants agreed that the artistic setting is an added value to the sessions because being in an aesthetically pleasing environment boosts the pleasantness of the activity. (Patient, female: “*You're distracted by looking at a painting, and the movement becomes wider, more natural, because you're not even thinking about doing the exercise*.”) (Patient, male: “*Because culture drives us, curiosity drives us. Beautiful things make us feel better.”*)
Frequency and Duration	Concerning the duration of the intervention, the proposed period was set at three months (from March to May), with a possible extension of an additional two months. All participants agreed that the timing of the intervention was convenient, especially since many people during the summer might not be able to attend the sessions. Regarding each individual session, participants preferred morning sessions over evening ones. Everyone agreed that 60 min per session is an ideal length.
Obstacles and facilitators	
Obstacles to participation	Participants primarily identified accessibility and parking spots as the main limitation of participation.
Facilitators for adherence	Regarding adherence, the diversity of the group (different ages, different motor difficulties) acted as a long-term incentive. Rather than a hindrance, the variation, making the intervention an asset for personal growth and future preparation. (Caregiver, female: “*No, it doesn't hold me back, on the contrary, it's all added value. I mean, knowing how things evolve, understanding how one woman experienced it, how another woman lived it, it's an extra asset.”*). Lastly, participants identified the prospect of peer connection as a crucial facilitator for adherence. Beyond mere social interaction, the opportunity to share space with those who have gone through similar experiences acted as a primary incentive reducing the hesitation often associated with starting a new intervention (Patient, female: “*I think one of the motivations could really be the idea of being together, being with people who have gone through the same journey*).

### Intervention phase

#### Study population

**Figure 1:**
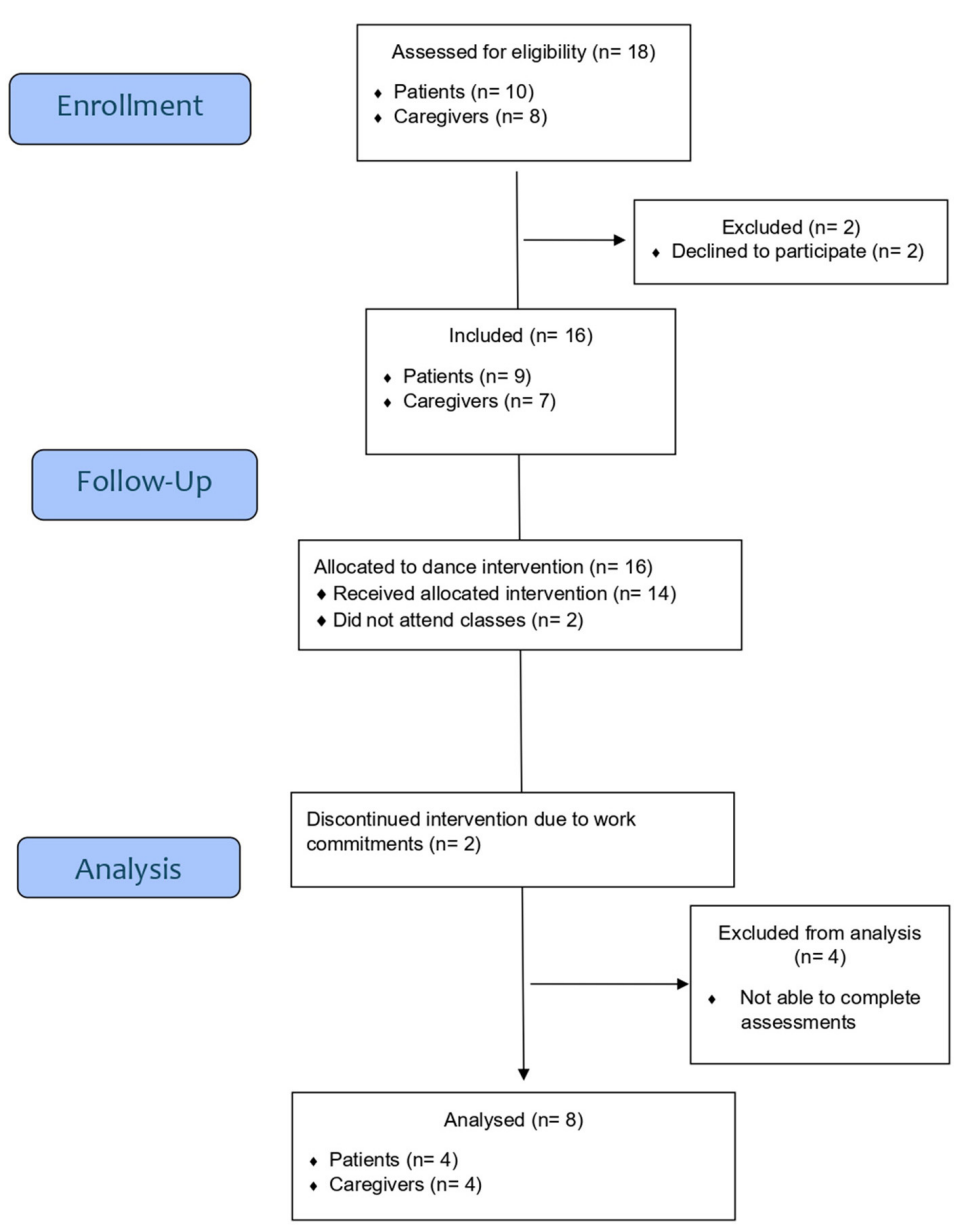
Consort 2010 flow diagram.

A Consolidated Standards of Reporting Trials (CONSORT) flow diagram for participant enrollment is shown in Figure 1. Initially, 10 patients and eight caregivers were screened for eligibility, with two declining to participate. Consequently, 16 participants were enrolled and allocated to the intervention (nine patients, seven caregivers). Of these, two participants (one patient, one caregiver) failed to attend any sessions. During the intervention, two caregivers dropped out due to work commitments. Of the remaining participants who completed the intervention, four patients were unable to complete the post-intervention assessments due to cognitive and motor limitations. The final analysis included four patients and four caregivers. Demographic and clinical characteristics are summarized in Table 2.

**Table 2:** Characteristics of participants that were included in the final analysis.

ID	Role	Age (*y*)	Sex	Education (*y*)	Stroke type	Months since stroke
P1	Patient	75	M	13	Hemorrhagic	10
P2	Patient	83	M	13	Ischemic	14
P3	Patient	86	F	10	Ischemic	8
P4	Patient	68	M	8	Ischemic	18
C1	Caregiver (wife)	62	F	13	—	—
C2	Caregiver (wife)	54	F	8	—	—
C3	Caregiver (wife)	73	F	13	—	—
C4	Caregiver (wife)	65	F	8	—	—

#### Feasibility

The retention rate was 85.7%, with 12 of the 14 enrolled participants completing the 20-week program. The two dropouts were caregivers who withdrew due to work-related time constraints. No stroke survivors withdrew from the study. The mean attendance rate for those who completed the intervention was 76%, indicating strong adherence to the dance sessions.

#### Adverse events and effects

There were no adverse events (e.g., falls, injuries) during the dance classes. Outside of the dance classes, one participant experienced a non-injurious fall, which was determined to be unrelated to the study intervention.

### Clinical outcomes

While 12 participants completed the intervention sessions (including those with severe aphasia and/or those utilizing wheelchairs), only eight of those participants took part in the post-intervention assessments. Therefore, the cognitive and psychosocial outcomes were explored descriptively in the four patients and four caregivers (see Table 3). In patients, mean score improvements were observed in global cognition (MoCA), Digit Span, and Short Story Recall, alongside modest reductions in completion times for the Trail Making Test (Parts A and B). Psychosocial wellbeing showed minimal changes with substantial individual variability. Caregivers demonstrated mean score increases in global cognition (MoCA), short story recall, and wellbeing (WHO-5), while caregiver burden remained largely stable. Given the very small sample size (*n* = 4 per group), *p*-values are reported for descriptive purposes only and do not indicate statistical evidence of effectiveness. Furthermore, while rank-biserial correlation (*r*_*c*_) estimates suggest magnitudes ranging from negligible to large, these should be interpreted with extreme caution due to the instability of effect size calculations in small samples.

**Table 3:** Cognitive and psychosocial outcomes.

Outcome	T0	T1	*Z*	*p*-value	Rank biserial correlation (*r*)
Patients (*n* = 4)					
Cognitive functions					
MoCA	22.5 (2.65)	25.5 (1.91)	−1.826	0.125	−1.000
TMT-A (sec.)	59.50 (20.14)	54.75 (19.99)	0.365	0.875	0.200
TMT-B (sec.)	223 (96.41)	209.5 (63.8)	0.365	0.875	0.200
DS Forward	6 (1.16)	6.50 (0.58)	−1.342	0.346	−1.000
DS Backward	4.00 (0.82)	4.25 (1.26)	−1.000	1.000	−1.000
Short story	7.83 (5.37)	10.55 (5.15)	−1.826	0.125	−1.000
Psychosocial wellbeing					
HADS–A	7.75 (4.57)	8.25 (5.62)	0.000	1.000	0.000
HADS–D	5.25 (4.03)	6.50 (5.80)	−0.548	0.713	−0.300
B-IPQ	56.00 (13.34)	54.75 (14.63)	0.000	1.000	0.000
**Caregivers (*****n*** = **4)**					
Cognitive functions					
MoCA	27.00 (1.41)	28.75 (1.89)	−1.826	0.098	−1.000
TMT-A (sec.)	43.25 (27.11)	43.25 (17.61)	−0.365	0.875	−0.200
TMT-B (sec.)	112.25 (49.26)	150.75 (81.29)	−1.461	0.250	−0.800
DS Forward	5.50 (0.57)	5.75 (0.50)	−0.535	0.773	−0.333
DS Backward	4.00 (0.00)	4.00 (0.00)	—^*a*^	—^*a*^	—^*a*^
Short story	11.78 (1.96)	15.15 (0.82)	−1.826	0.125	−1.000
Psychosocial wellbeing					
WHO-5	11.25 (4.11)	14.00 (3.37)	−1.826	0.098	−1.000
Zarit burden interview	31.75 (10.99)	30.75 (18.01)	0.183	1.000	0.100

Cognitive and psychosocial outcomes were analyzed using Wilcoxon signed-rank test. Pre and post-test values presented are means and standard deviations. Z = Standardized test for the Wilcoxon signed-rank test.

Effect size is reported using rank-biserial correlation (r), negative values indicate higher post-test scores relative to pre-test.

^*a*^Statistical comparison was not possible for Digit Span Backward due to zero variance in scores (all participants scored 4.0 at both time points).

MoCA, montreal cognitive assessment; TMT-A, trail making test part A; TMT-B, trail making test part B; DS, digit span; HADS, Hospital Anxiety and Depression Scale; B-IPQ, Brief Illness Perception Questionnaire; WHO-5, World Health Organization Wellbeing Index.

Motor outcomes are reported in Table 4. Among patients, mean increases were observed in gait velocity and cadence, while step length and stride length showed reductions accompanied by decreased variability at post-test. A marked reduction in gait asymmetry was observed at the group level, although substantial inter-individual variability was present at baseline. Balance measures indicated reductions in total sway length, velocity, and sway area under both eyes-open and eyes-closed conditions, with greater variability observed in the eyes-closed condition. In contrast, the gait parameters of caregivers remained largely stable across assessments, with small changes in mean values and increased variability for cadence at post-test. Balance outcomes in caregivers also showed limited mean changes, although sway area demonstrated substantial inter-individual variability, particularly under the eyes-closed condition.

**Table 4:** Motor outcomes.

Measure	T0	T1	*Z*	*p*-value	Rank biserial correlation (*r*)
Patients (*n* = 4)					
Spatiotemporal gait					
Velocity (m/s)	0.55 (0.27)	0.61 (0.24)	−1.461	0.250	−0.800
Step length (cm)	57.05 (12.25)	50.45 (6.88)	1.826	0.125	1.000
Stride length (cm)	111.35 (25.89)	99.85 (12.95)	1.826	0.125	1.000
Cadence (steps/min)	32.99 (15.09)	37.81 (18.08)	−1.095	0.375	−0.600
Imbalance (%)	−78.87 (61.59)	−1.31 (14.09)	−0.730	0.625	−0.400
**Balance**					
Eyes open					
Total length (mm)	561.64 (192.92)	519.44 (202.90)	0.000	1.000	0.000
Total velocity (mm/s)	18.72 (6.43)	17.32 (6.76)	0.000	1.000	0.000
Sway area (mm^2^)	596.52 (798.32)	288.02 (172.38)	−0.535	0.750	−0.333
Eyes close					
Total length (mm)	916.02 (233.21)	717.03 (248.76)	1.826	0.125	1.000
Total velocity (mm/s)	30.53 (7.77)	23.90 (8.29)	1.826	0.125	1.000
Sway area (mm^2^)	906.86 (1,063.44)	300.26 (295.28)	1.826	0.125	1.000
**Caregivers (*****n*** = **4)**					
Spatiotemporal gait					
Velocity (m/s)	1.03 (0.18)	0.99 (0.13)	0.730	0.625	0.400
Step length (cm)	56.03 (2.09)	56.73 (5.43)	−0.365	0.875	−0.200
Stride length (cm)	113.75 (10.75)	113.85 (10.75)	0.000	1.000	0.000
Cadence (steps/min)	54.13 (6.47)	65.73 (27.07)	0.000	1.000	0.000
Imbalance (%)	−1.93 (9.35)	−0.35 (3.25)	0.365	0.875	0.200
**Balance**					
Eyes open					
Total length	227.64 (33.29)	221.18 (25.78)	0.000	1.000	0.000
Total velocity (mm/s)	7.58 (1.11)	7.37 (0.86)	0.000	1.000	0.000
Sway area (mm^2^)	157.85 (100.29)	176.41 (137.69)	−0.535	0.750	−0.333
Eyes closed					
Total length (mm)	301.48 (38.43)	281.92 (41.18)	0.535	0.750	0.333
Total velocity (mm/s)	10.05 (1.28)	9.39 (1.37)	0.535	0.750	0.333
Sway area (mm^2^)	212.22 (127.33)	163.88 (114.69)	1.069	0.500	0.677

Motor outcomes were analyzed using Wilcoxon signed-rank test. Pre and post-test values presented are means and standard deviations.

Z = Standardized test for the Wilcoxon signed-rank test. Effect size is reported using rank-biserial correlation (r), negative values indicate higher post-test scores relative to pre-test.

#### Exit Questionnaire

Participants reported a positive experience and expressed interest in continuing if more classes were offered. They enjoyed meeting new people and feeling a sense of community with others who had experienced a stroke, as well as with caregivers whose partners had experienced a stroke. In addition to the social benefits, participants reported moderate improvements in their physical and emotional health (see Table 5). They also praised the dance instructors' expertise and motivational approach. Overall, participants rated the program as feasible.

**Table 5:** Participant responses to Exit Questionnaire.

Questionnaire item	Median (1st, 3rd quartiles)
I enjoyed the program.	1 (1.00, 1.25)
My balance has improved.	2 (1.75, 3.00)
My walking has improved.	2 (1.75, 2.25)
My mood has improved.	2 (1.00, 3.00)
My coordination has improved.	2 (1.00, 2.25)
My strength has improved.	2 (1.75, 3.00)
My endurance has improved.	2 (1.75, 2.25)
If I could, I would continue the program.	1.5 (1.00, 2.00)
I use the skills I learned in class in daily life.	2 (2.00, 2.00)
The program is feasible in overall.	1 (1.00, 1.25)

Participants rated each item on the Exit Questionnaire on a scale of one to five. 1, Strongly agree, 2, Somewhat agree, 3, Neither agree nor disagree, 4, Somewhat disagree, 5, Strongly disagree.

## Discussion

The present study demonstrates that a 20-week co-created contemporary dance intervention is a feasible, safe, and inclusive program for stroke survivors and their caregivers. While the intervention's core components were established *a priori*, the focus group discussion was essential to validate logistical choices and identify barriers to adherence. Specifically, aligning the sessions with participants' preferences for morning hours and concluding before the August holiday period ensured the program met the community's constraints. This congruence likely contributed to the high retention and attendance rates observed.

Unlike traditional clinical practice, our multidimensional model integrated physical, cognitive, and social engagement without adverse events. Notably, participants who could not complete standardized gait or cognitive assessments were still fully engaged in the dance sessions. This highlights contemporary dance as a more accessible rehabilitation alternative for severely impaired survivors who might otherwise be excluded from traditional physical activity programs. In addition, conducting sessions in a historic, culturally rich environment provided an artistic and socially inclusive atmosphere that intentionally moved away from a sterile clinical rehabilitation setting, which participants found highly motivating. Specifically, because the venue remained open to the public, it transformed the experience from an isolated clinical routine into a shared activity, fostering social inclusion by connecting participants directly with the local community. Furthermore, immersing the sessions within a cultural heritage site underscores the vital role of art as a form of creative stimulation. When sessions were held in the historic fresco hall, participants directly interacted with the artwork through movement, using the visual art to express emotions and shape their bodies in response to the surrounding masterpieces. Providing evidence for the multimodal impacts of arts-in-health initiatives, this direct embodiment of the cultural space acts as an active supportive component, which has been shown to enhance emotional resonance and promote a sense of wellbeing and belonging (Fancourt and Finn, 2019).

The inclusion of informal caregivers aligns with evidence supporting dyadic approaches that engage both patients and their caregivers in shared rehabilitation activities. Previous studies suggest that caregiver involvement can enhance rehabilitation engagement, support continuity of practice in daily life, and benefit psychosocial outcomes for both stroke survivors and caregivers (Doyle et al., 2021). Dyadic interventions targeting the patient–caregiver relationship have been associated with reduced caregiver burden, improved emotional adjustment, and increased participation in rehabilitation activities (Kazemi et al., 2021). Similarly, psychosocial support programs highlight the importance of addressing caregiver stress, coping strategies, and role adaptation following a stroke (Zhang et al., 2023). Within this context, the present study extends existing work by directly involving informal caregivers in a dance intervention designed to engage both members of the dyad simultaneously. This approach may complement established rehabilitation models by integrating the physical, cognitive, and relational dimensions of recovery.

### Feasibility and acceptability

Recruitment challenges resulted in an initial cohort of 16 participants (with 14 starting the intervention), which was below the target of 24. However, the 85.7% completion rate (12/14) and participants' desire to continue indicate high acceptability and commitment. Notably, the smaller group size facilitated a lower instructor-to-participant ratio, allowing for greater individual attention. This increased personalization likely reinforced participant motivation and contributed to the positive trends in psychosocial outcomes observed. In line with previous research (Demers et al., 2015; Swaine et al., 2020), our participants reported that they enjoyed the sessions, appreciated the social interaction, and would continue if future sessions were offered.

### Clinical implications

In this exploratory sample, patients showed positive trends in global cognition (MoCA) and long-term memory (Short Story Recall). In addition, we observed a trend toward reduced completion times for processing speed and visual attention tasks (TMT-A and TMT-B). These findings align with previous research indicating that the dual-task nature of dance, combining physical activity with rhythmic and sequence learning, holds the potential to support cognitive plasticity following a stroke (Teixeira-Machado et al., 2019). Interestingly, similar positive trends in global cognition and memory were observed among caregivers. The dance intervention may have served as a form of social enrichment for caregivers, which has been shown to promote neuroplasticity and cognitive reserve in older adults (Kelly et al., 2017). While patient psychosocial outcomes remained stable, caregivers reported an encouraging increase in overall wellbeing. Previous research suggests that for caregivers, the transition from a purely task-oriented caregiving role to a shared social activity can positively influence quality of life (Vloothuis et al., 2019). Notably, subjective caregiver burden (ZBI) remained unaltered, which may suggest that while the shared activity improves mood, the logistical and physical demands of daily caregiving are not fully mitigated by a weekly intervention. The ZBI assesses domains such as financial strain, the patient's physical dependency, and the long-term impact on the caregiver's health and social life, factors that are outside the scope of a dance intervention. Regarding motor outcomes, patients showed increased gait velocity and cadence within this small group. An encouraging exploratory observation was the reduction in gait asymmetry, which is typically pronounced in hemiparetic stroke survivors (Balasubramanian et al., 2007). In contrast, caregiver gait and balance measures remained stable, suggesting that the intervention's physical intensity was sufficient for stroke rehabilitation but acted primarily as a maintenance activity for the healthy caregivers.

### Study limitations

This feasibility study included a small sample that limits the statistical power and the generalizability of the findings to the broader stroke and caregiver populations. All post-stroke participants were in the chronic stage of recovery; therefore, the effectiveness and safety of a contemporary dance intervention in acute or subacute patients were not tested. An additional limitation concerns the discrepancy between intervention participation and outcome assessment completion. Although all participants completed the intervention, only four stroke survivors were able to complete the full assessment battery due to cognitive and/or motor impairments. As a result, the quantitative analyses may reflect a higher-functioning subgroup of stroke survivors, introducing a potential selection bias. Consequently, the observed changes in cognitive and motor outcomes should be interpreted cautiously, as they may not be representative of the entire sample of participants who engaged in the intervention. This also highlights a potential mismatch between the accessibility of the intervention, which was feasible for individuals with more severe impairments, and the feasibility of administering standardized outcome measures in this population. Another limitation relates to the interpretation of the cognitive outcomes. Although a 5-month interval between baseline and post-intervention assessments reduces the likelihood of practice effects, these cannot be fully excluded given the repeated administration of identical instruments (e.g., MoCA and Trail Making Test). Participants may still show improved performance due to increased familiarity with test procedures or reduced test-related anxiety. Therefore, the observed cognitive improvements should be interpreted cautiously and considered exploratory, especially in the absence of a control group.

### Future research

To facilitate a larger-scale intervention, several changes and adjustments to the study procedure are recommended. Specifically, recruitment challenges observed in the present study highlight important considerations for future definitive trials. Engaging stroke survivors with varying levels of physical and cognitive impairment, as well as their informal caregivers, requires tailored recruitment strategies involving collaboration with clinical services, community organizations, and caregiver networks. Future studies may benefit from structured referral routes through stroke rehabilitation services and neurology clinics, as well as engagement with community-based and caregiver support organizations to facilitate access to eligible participants and improve recruitment efficiency. In addition, recruitment materials specifically addressing caregivers may improve enrollment, given their key role in facilitating participation in dyadic interventions. Logistical factors such as transportation support, flexible scheduling, and accessible venue selection may further enhance enrollment and retention. Taken together, these strategies are likely to improve feasibility and representativeness in larger-scale trials of dyadic and dance-based interventions. Beyond recruitment, the feasibility of the intervention's content and structure offers critical insights into program revision. Regarding what worked, the open-ended nature of contemporary dance provided an ideal framework for inclusivity, allowing participants to adjust the movements to their own comfort levels. A primary facilitator of this success was the high level of specialization of the dance teachers, who possessed over 5 years of experience working with neurological populations, specifically with Parkinson's disease. Furthermore, the active presence of informal caregivers served as a powerful facilitator: namely, their emotional support and practical encouragement directly motivated the stroke survivors to attend regularly, resulting in higher participant retention. Conversely, an aspect of the program that did not work optimally for everyone was the scheduling of sessions during morning hours, which created a significant barrier for working caregivers who could not attend. Therefore, future program adaptations should consider offering more flexible scheduling (such as late afternoon or weekend sessions) to better accommodate working families. In addition, a Randomized Controlled Trial (RCT) comparing this patient-caregiver dance intervention to standard rehabilitation is necessary to isolate whether the benefits stem from physical movement, social interaction, or the shared patient-caregiver experience.

## Conclusions

This study suggests that a 20-week contemporary dance intervention, developed through a co-creation design, is a feasible, safe, and highly acceptable community approach for stroke survivors and their caregivers. Despite the challenges of recruiting, high retention and positive trends in patient cognition and caregiver wellbeing were observed. Therefore, our study suggests that co-created contemporary dance programs offer not only a complementary community-based approach to post-stroke rehabilitation, but also a dynamic, movement-based adjunct to traditional support groups for informal caregivers.

## Data Availability

The raw data supporting the conclusions of this article will be made available by the authors, without undue reservation.

## References

[B1] Balasubramanian C. K. Bowden M. G. Neptune R. R. Kautz S. A. (2007). Relationship between step length asymmetry and walking performance in subjects with chronic hemiparesis. Arch. Phys. Med. Rehabil. 88, 43–49. doi: 10.1016/j.apmr.2006.10.004 17207674

[B2] Bensing J. Rimondini M. Visser A. (2013). What patients want. Patient Educ. Couns. 90, 287–90. doi: 10.1016/j.pec.2013.01.005 23395286

[B3] Bonomi A. E. Patrick D. L. Bushnell D. M. Martin M. (2000). Validation of the United States' version of the world health organization quality of life (WHOQOL) instrument. J. Clin. Epidemiol.53, 1–12. doi: 10.1016/S0895-4356(99)00123-7 10693897

[B4] Broadbent E. Petrie K. J. Main J. Weinman J. (2006). The brief illness perception questionnaire. J. Psychosom Res. 60, 631–7. doi: 10.1016/j.jpsychores.2005.10.020 16731240

[B5] Chattat R. Cortesi V. Izzicupo F. Del Re M. L. Sgarbi C. Fabbo A. . (2011). The Italian version of the Zarit Burden interview: a validation study. Int. Psychogeriatr. 23, 797–805. doi: 10.1017/S1041610210002218 21205379

[B6] Comune di Bassano del Grappa. (2026). The project. Dance Well,–Movement Research for Parkinson. Available online at: https://dancewell.eu/en/the-project (Accessed January 13, 2026).

[B7] Costantini M. Musso M. Viterbori P. Bonci F. Del Mastro L. Garrone O. . (1999). Detecting psychological distress in cancer patients: validity of the Italian version of the hospital anxiety and depression Scale. Support Care Cancer. 7, 121–127. doi: 10.1007/s005200050241 10335929

[B8] Crowe C. Coen R. F. Kidd N. Hevey D. Cooney J. Harbison J. (2016). A qualitative study of the experience of psychological distress post-stroke. J. Health Psychol. 21, 2572–9. doi: 10.1177/1359105315581067 25930078

[B9] Demers M. Thomas A. Wittich W. McKinley P. (2015). Implementing a novel dance intervention in rehabilitation: perceived barriers and facilitators. Disabil. Rehabil. 37, 1066–1072. doi: 10.3109/09638288.2014.955135 25163831

[B10] Donisi V. Poli S. Mazzi M. A. Gobbin F. Schena F. Del Piccolo L. . (2022). Promoting participatory research in chronicity: the ESPRIMO biopsychosocial intervention for young adults with multiple sclerosis. Front Psychol. 13:1042234., doi: 10.3389/fpsyg.2022.1042234 36405126 PMC9669711

[B11] Doyle K. L. Toepfer M. Bradfield A. F. Noffke A. Ausderau K. K. Andreae S. . (2021). Systematic review of exercise for caregiver–care recipient dyads: what is best for spousal caregivers—exercising together or not at all?. Gerontologist.61, *e*283–e301. doi: 10.1093/geront/gnaa043 32614050 PMC8361501

[B12] Fancourt D. Finn S. (2019). What is the Evidence on the Role of the Arts in Improving Health and Well-Being? A Scoping Review. World Health Organization Regional Office for Europe. 14–17. 32091683

[B13] Feigin V. L. Abajobir A. A. Abate K. H. Abd-Allah F. Abdulle A. M. Abera S. F. . (2017). Global, regional, and national burden of neurological disorders during 1990–2015: a systematic analysis for the global burden of disease study 2015. Lancet Neurol. 16, 877–897. doi: 10.1016/S1474-4422(17)30299-5 28931491 PMC5641502

[B14] Foster P. P. (2013). How does dancing promote brain reconditioning in the elderly? Front. Aging Neurosci. 5:4. doi: 10.3389/fnagi.2013.00004 23447744 PMC3581818

[B15] Hackney M. E. Earhart G. M. (2009). Effects of dance on movement control in Parkinson's disease: a comparison of *Argentine tango* and American ballroom. J. Rehabil. Med. 41, 475–81. doi: 10.2340/16501977-0362 19479161 PMC2688709

[B16] Hackney M. E. Hall C. D. Echt K. V. Wolf S. L. (2012). Application of adapted tango as therapeutic intervention for patients with chronic stroke. J. Geriatr. Phys. Ther. 35, 206–17. doi: 10.1519/JPT.0b013e31823ae6ea 22143597

[B17] Hackney M. E. Kantorovich S. Earhart G. M. (2007). A study on the effects of Argentine tango as a form of partnered dance for those with Parkinson disease and the healthy elderly. Am. J. Dance Ther. 29, 109–127., doi: 10.1007/s10465-007-9039-2

[B18] Hsieh H. F. Shannon S. E. (2005). Three approaches to qualitative content analysis. Qual. Health Res.15, 1277–88. doi: 10.1177/1049732305276687 16204405

[B19] Jokinen H. Melkas S. Ylikoski R. Pohjasvaara T. Kaste M. Erkinjuntti T. . (2015). Post-stroke cognitive impairment is common even after successful clinical recovery. Eur. J. Neurol. 22, 1288–94. doi: 10.1111/ene.12743 26040251

[B20] Kattenstroth J. C. Kolankowska I. Kalisch T. Dinse H. R. (2010). Superior sensory, motor, and cognitive performance in elderly individuals with multi-year dancing activities. Front. Aging Neurosci. 2:31. doi: 10.3389/fnagi.2010.00031 20725636 PMC2917240

[B21] Kazemi A. Azimian J. Mafi M. Allen K. A. Motalebi S. A. (2021). Caregiver burden and coping strategies in caregivers of older patients with stroke. BMC Psychol. 9:51. doi: 10.1186/s40359-021-00556-z 33794995 PMC8017750

[B22] Kelly M. E. Duff H. Kelly S. McHugh Power J. E. Brennan S. Lawlor B. A. . (2017). The impact of social activities, social networks and social support on cognitive functioning in older adults: a systematic review. Exp. Gerontol. 94, 103–114., doi: 10.1186/s13643-017-0632-2 29258596 PMC5735742

[B23] Keogh J. W. Kilding A. Pidgeon P. Ashley L. Gillis D. . (2009). Physical benefits of dancing for healthy older adults: a review. J. Aging Phys. Act. 17, 479–500. doi: 10.1123/japa.17.4.479 19940326

[B24] Kipnis D. Kruusamäe H. King M. Schreier A. R. Quinn L. Shih H.-J. S. . (2023). Dance interventions for individuals post-stroke—a scoping review. Top Stroke Rehabil. 30, 768–85. doi: 10.1080/10749357.2022.2107469 35968809

[B25] Klimova B. Valis M. Kuca K. (2017). Dancing as an intervention tool for people with dementia: a mini-review dancing and dementia. Curr Alzheimer Res. 14, 1264–9. doi: 10.2174/1567205014666170713161422 28714391

[B26] Langhorne P. Bernhardt J. Kwakkel G. (2011). Stroke rehabilitation. Lancet. 377, 1693–1702. doi: 10.1016/S0140-6736(11)60325-5 21571152

[B27] Langhorne P. Coupar F. Pollock A. (2009). Motor recovery after stroke: a systematic review. Lancet Neurol. 8, 741–754. doi: 10.1016/S1474-4422(09)70150-4 19608100

[B28] Li X. He Y. Wang D. Rezaei M. J. (2024). Stroke rehabilitation: from diagnosis to therapy. Front. Neurol. 15:1402729. doi: 10.3389/fneur.2024.1402729 39193145 PMC11347453

[B29] Liu X. Shen P. L. Tsai Y. S. (2021). Dance intervention effects on physical function in healthy older adults: a systematic review and meta-analysis. Aging Clin. Exp. Res. 33, 253–263. doi: 10.1007/s40520-019-01440-y 31894561

[B30] Monaco M. Costa A. Caltagirone C. Carlesimo G. A. (2013). Forward and backward span for verbal and visuo-spatial data: standardization and normative data from an Italian adult population. Neurol Sci. 34, 749–754. doi: 10.1007/s10072-012-1130-x 22689311

[B31] Mukherjee D. Levin R. L. Heller W. (2006). The cognitive, emotional, and social sequelae of stroke: psychological and ethical concerns in post-stroke adaptation. Top Stroke Rehabil. 13, 26–35. doi: 10.1310/tsr1304-26 17082166

[B32] Pain D. Miglioretti M. Angelino E. (2006). Sviluppo della versione italiana del Brief-IPQ, strumento psicometrico per lo studio delle rappresentazioni di malattia. Psicologia della Salute 2006, 81–89. doi: 10.1400/91143

[B33] Rigby H. Gubitz G. Phillips S. (2009). A systematic review of caregiver burden following stroke. Int. J. Stroke. 4, 285–92. doi: 10.1111/j.1747-4949.2009.00289.x 19689757

[B34] Rochetti L. M. de Assis I. S. A. Caires T. A. Emílio M. M. de Almeida R. O. de Souza L. A. P. A. . (2021). Effects of Bolero basic steps on balance and functional mobility in post-stroke hemiparesis: a pilot study. J Bodyw Mov Ther. 25, 188–192. doi: 10.1016/j.jbmt.2020.10.016 33714494

[B35] Rost N. S. Brodtmann A. Pase M. P. van Veluw S. J. Biffi A. Duering M. Hinman J. D. . (2022). Post-stroke cognitive impairment and. Circ Res. 130, 1252–1271. doi: 10.1161/CIRCRESAHA.122.319951 35420911

[B36] Salter K. L. Hellings C. Foley N. C. Teasell R. W. (2008). The experience of living with stroke: a qualitative meta-synthesis. J. Rehabil. Med. 40, 595–602. doi: 10.2340/16501977-0238 19020691

[B37] Salvadori E. Pasi M. Poggesi A. Chiti G. Inzitari D. Pantoni L. (2013). Predictive value of MoCA in the acute phase of stroke on the diagnosis of mid-term cognitive impairment. J Neurol. 260, 2220–2227. doi: 10.1007/s00415-013-6962-7 23716072

[B38] Schmid A. A. Van Puymbroeck M. Altenburger P. A. Miller K. K. Combs S. A. Page S. J. (2013). Balance is associated with quality of life in chronic stroke. Top Stroke Rehabil. 20, 340–6. doi: 10.1310/tsr2004-340 23893833

[B39] Siciliano M. Chiorri C. Battini V. Sant'Elia V. Altieri M. Trojano L. . (2019). Regression-based normative data and equivalent scores for Trail Making Test (TMT): an updated Italian normative study. Neurol Sci. 40, 469–477. doi: 10.1007/s10072-018-3673-y 30535956

[B40] Siciliano M. Chiorri C. Passaniti C. Sant'Elia V. Trojano L. Santangelo G. . (2019). Comparison of alternate and original forms of the montreal cognitive assessment (MoCA): an Italian normative study. Neurol Sci. 40, 691–702. doi: 10.1007/s10072-019-3700-7 30637545

[B41] Spinnler H. and Tognoni, G. (1987) Standardizzazione e taratura italiana di test neuropsicologici. Ital. J. Neurol. Sci. 8, 44–46. 10.7551/mitpress/4635.001.0001.

[B42] Swaine B. Poncet F. Lachance B. Proulx-Goulet C. Bergeron V. Brousse É. . (2020). The effectiveness of dance therapy as an adjunct to rehabilitation of adults with a physical disability. Front. Psychol. 11:1963. doi: 10.3389/fpsyg.2020.01963 32982831 PMC7479122

[B43] Teixeira-Machado L. Arida R. M. Jesus Mari de. J. (2019). Dance for neuroplasticity: a descriptive systematic review. Neurosci. Biobehav. Rev. 96, 232–240., doi: 10.1016/j.neubiorev.2018.12.010 30543905

[B44] Varga L. B. Benda C. Nagy E. (2023). The benefits of enhanced folk dance sessions for stroke rehabilitation in terms of motor learning. Rehabil. Med. 27, 49–55. doi: 10.5604/01.3001.0054.2773

[B45] Velloze I. G. Jester D. J. Jeste D. V. Mausbach B. T. (2022). Interventions to reduce loneliness in caregivers: an integrative review of the literature. Psychiatry Res. 311:114508., doi: 10.1016/j.psychres.2022.114508 35334424

[B46] Vloothuis J. D. Mulder M. Nijland R. H. Goedhart Q. S. Konijnenbelt M. Mulder H. . (2019). Caregiver-mediated exercises with e-health support for early supported discharge after stroke (CARE4STROKE): a randomized controlled trial. PloS ONE 14:e0214241. doi: 10.1371/journal.pone.0214241 30958833 PMC6453481

[B47] Volpe D. Baldassarre M. G. Bakdounes L. Campo M. C. Ferrazzoli D. Ortelli P. (2025). “Dance well”—a multisensory artistic dance intervention for people with parkinson's disease: a pilot study and brain. Sciences. 15:357. doi: 10.3390/brainsci15040357 PMC12025761 40309840

[B48] Wagle J. Farner L. Flekkøy K. Bruun Wyller T. Sandvik L. Fure B. . (2011). Early post-stroke cognition in stroke rehabilitation patients predicts functional outcome at 13 months. Dement. Geriatr. Cogn. Disord. 31, 379–87. doi: 10.1159/000328970 21720162

[B49] World Medical Association and World Medical Association. (2025). Declaration of Helsinki: ethical principles for medical research involving human participants. JAMA. 333, 71–4. doi: 10.1001/jama.2024.21972 39425955

[B50] Zarit S. H. Edwards A. B. (1996). Family caregiving: research and clinical intervention. In: Woods RT, editor. Handbook of the Clinical Psychology of Ageing. 2nd ed. Chichester: John Wiley and Sons. p. 331–368.

[B51] Zhang X. Y. Sha K. H. Ma X. Y. Li X. M. Zhang M. H. (2023). Dyadic psycho-social interventions for stroke survivors and family caregivers: A systematic review and meta-analysis of randomized controlled trials. J. Adv. Nurs. 79, 3707–3726. doi: 10.1111/jan.15781 37439492

[B52] Zigmond A. S. Snaith R. P. (1983). The hospital anxiety and depression scale. Acta Psychiatr Scand. 67, 361–70. doi: 10.1111/j.1600-0447.1983.tb09716.x 6880820

