# Supplementary material for: Dance intervention targeting motor, cognitive, and emotional components in stroke patients and their caregivers: a feasibility study
Source: Front Psychol. 2026 Jul 27;17:1872042. doi: 10.3389/fpsyg.2026.1872042 (PMC13454283; doi:10.3389/fpsyg.2026.1872042)
Supplement: Supplementary file 1 [file Supplementary_file_1.docx]

**Supplementary material**

**Supplementary appendix 1 - List of the open-ended questions used in the focus group discussion**

1.) What are the main objectives that a group intervention should have to promote the well-being of the post-stroke population and their caregivers?
2.) What would you like to find in these meetings from a psychological, motor, and social perspective?
3.) From a socio-relational perspective, what needs would you like to be addressed in an intervention?
4.) How do you feel about the venue (museum) where the intervention will take place?
5.) What could encourage people to participate in this intervention?
6.) What could be a barrier to taking part in these meetings?
7.) What actions could motivate/encourage people to participate?

**Supplementary appendix 2 – Intervention phase**

Part of the dance strategy is using cultural spaces, such as museums and galleries to promote dancing in artistic environments. Therefore, the intervention took place at a museum (*Museo degli Affreschi “G.B. Cavalcaselle” alla Tomba di Giulietta*) in Verona, Italy. The presence of frescoes provided significant aesthetic value to the environment. Furthermore, participants engaged in social interaction before and after sessions, adding a distinct social dimension to the intervention. Each session began with participants seated in a circle, focusing on breathwork and transitioning gradually from sitting to standing through limb-stretching exercises. This was followed by a phase of improvisation and creative play, where participants were encouraged to adapt movements to overcome physical limitations. Subsequently, participants learned a choreographed sequence set to music and collaborated on a group piece. Lastly, each session concluded in a circle, where participants performed a personalized gesture to close.

Distinctive features of the dance sessions

1) Stimulating environment: dancing among the works of art in a museum, with appropriate guidance during the sessions, encourages creativity, beauty, and free expression.

2) The choice of music: the music accompanying the sessions stimulates coordination and supports the images and sensations offered to participants, following specific contents which connect the meetings.

3) Physical contact: for people whose daily lives have changed dramatically due to illness, rediscovering physical contact between bodies, even with strangers, contributes to socialization and empathy.

4) Socialization and inclusion: participants find themselves in a welcoming environment that helps them socialize and form new relationships, even beyond the dance sessions, where inclusion remains a central element. Participants find themselves in an environment where their possibilities are emphasized rather than their limitations.

5) Natural environment: weather permitting, dance sessions take place in the museum park, where nature helps to create a sense of well-being.

6) Public place: museum visitors can observe the dance sessions and sometimes take part in them. The actions of the regular participants of the dance sessions acquire therefore public significance, creating a sense of belonging to a community.

**Supplementary Table S1 - Characteristics of the focus group participants**

| **Characteristics** | **Stroke survivors (n =4)** | **Caregivers (n=3) *** |
| --- | --- | --- |
| Age (*M*, years) | 76.8 (64-86) | 62.7 (54-73) |
| Gender |  |  |
| Female | 1 | 3 |
| Male | 3 | 0 |
| Marital status |  |  |
| Married | 3 | 3 |
| Widowed | 1 | 0 |
| Highest educational level |  |  |
| Secondary School | 1 | 1 |
| High School | 3 | 2 |
| Occupation |  |  |
| Retired | 4 | 2 |
| Other | 0 | 1 |
| Stroke type |  |  |
| Ischemic stroke | 2 | NR |
| Haemorrhagic stroke | 2 | NR |
| Stroke onset |  |  |
| >6 months | 4 | NR |

*NR,* not relevant.

*Caregivers are wives of three patients
